# Magnetic integrated double-trap filter utilizing the mutual inductance for reducing current harmonics in high-speed railway traction inverters

**DOI:** 10.1038/s41598-024-60877-y

**Published:** 2024-05-02

**Authors:** Maged Al-Barashi, Yongjun Wang, Bin Lan, Muhammad Shoaib Bhutta

**Affiliations:** 1 School of Aeronautics and Astronautics, Guilin University of Aerospace Technology, Guilin, 541004 China; 2 School of Automobile Engineering, Guilin University of Aerospace Technology, Guilin, 541004 China

**Keywords:** Engineering, Electrical and electronic engineering, Energy infrastructure, Energy science and technology, Energy infrastructure, Energy storage, Renewable energy

## Abstract

Current harmonics are generated at the switching frequency and its multiples when the traction converters are modulated. To address this, multi-trap filters are introduced, which are capable of selectively eliminating these specific harmonics to the limits set by IEEE 519-2014. This targeted removal significantly reduces the need for high total inductance, thereby allowing for a more compact filter design. Comparatively, to traditional inductor-capacitor-inductor (*LCL*) filters, more magnetic cores are needed for trap inductors. Furthermore, the traction systems have not been examined in conjunction with multi-trap filters. To reduce the filter size and investigate its application in traction converters, this paper presents an integrated double-trap *LCL* (*DTLCL*) filter. A tiny capacitor is connected in parallel with the grid-side inductor to form one *LC*-trap. In addition, another *LC*-trap is formed by connecting the equivalent trap inductor, introduced through the magnetic coupling between inverter-side and grid-side inductors, in series with the filter capacitor. The presented filters' features are thoroughly analyzed, and the design method has been developed. Finally, the simulation and hardware-in-the-loop (HIL) experiment results validate the proposed method's viability and efficacy. Compared to the discrete windings, the integrated ones enable a size decrease of two cores. Furthermore, the proposed filters can meet IEEE 519-2014 criteria with 0.3% for all the current switching harmonics and total harmonic distortion (THD) of 2.36% of the grid‐side current.

## Introduction

Electric multiple units (EMUs) with pulse width modulation (PWM) traction inverters are often used on China's high-speed railway (HSR) lines^1^. For modern railway traction power-supply systems (TPSS), the undesired current harmonics produced by the modulating of traction inverters are a well-known issue^2^. These harmonics might result in a variety of issues that decrease the effectiveness of the traction networks, including severe faults and interruptions, torque bursts, and communications network disruptions^3^. In several electrical railways, harmonic resonance occurrences have led to the breakdown of high-voltage facilities, the improper initiation of protective systems, or perhaps locomotive traction bottlenecks^4^. Therefore, a major issue for HSR lines is mitigating and addressing the harmonic distortion. The high-frequency harmonics, especially at the switching frequency and its multiples, are attenuated utilizing passive filters^5^, although the harmonics at low frequencies might be mitigated by properly designed repeated controllers^6^ or proportional-resonant ones^7^. Whenever tied to the TPSS, traction inverters must adhere to the network regulations.

The high-frequency harmonics problem in HSR is frequently addressed using either the traction electric grid or the high-speed train (HST) drive units. The traction electric grid's harmonics reduction has received the majority of research attention. To change the harmonic impedance in the traction electrical network, several studies employ passive filters^5^. Capacitor (*C*) and inductor-capacitor (*LC*) filters are examples of these filters. The passive filter method becomes quite costly for TPSS with high voltage and large power. From the perspective of the HST drive unit, there exists an alternative way to reduce the harmonics generated by the traction PWM inverters. This way attenuates the HSR high-frequency harmonics by using the selective harmonic elimination (SHE) PWM mechanism in the traction inverter^4^. It has become clear that the offline optimal PWM approach had a low tolerance to the system parameters and required complex calculations to solve the basic equations. High-frequency harmonics may be easily absorbed with large filter inductors. Even yet, a straightforward approach such as this may essentially lead to an increase in the system's size, costs, and control bandwidths. Excluding a tiny additional capacitor utilized in the technique given in^8^, the well-known third-order *LCL* filters effectively replaced the conventional *L* ones with an identical overall inductance value. Nevertheless, it is hard to attain a wide control bandwidth, high power density, and good harmonic removal capacity, even with the *LCL* filter^9^.

Over the past few years, grid-connected inverters have increasingly used *LCL* filters to minimize high-frequency harmonics in the input or output current of renewable power plants^10^. The standard *L*-type boost inverter, in contrast, has been routinely utilized by the HSTs, particularly as a grid-side inverter. To satisfy the requirements for small size and light weight in HST systems, the traction transformer (TT) leakage inductor is often employed as an inductor of the grid-side inverter^11^. Several *LCL*-modified filter topologies are used in^12–23^. Several of those filters also include extra *LC* traps, which are resonant tanks that are parallel or in series, and resonate with the switching frequency and its multiples. Such traps may be used to bypass or block certain harmonics. Consequently, such harmonics are omitted from the current delivered into the network, the total harmonic distortion (THD) is reduced, and the capacitance and inductance of the filters are decreased.

When utilizing multiple traps that resonate at the switching frequency and its multiples, the harmonics eliminated by the trap-filters may be enhanced, and associated inductances can be reduced. Examples of these filters are *LTL*^12,13,24,25^, *LTCL*^23,26–28^, *SPRLCL*^9^, *L*(*LCL*)_2_^29^, and *PDTLCL*^30^ filters, in which the letter "T*"* denotes one or more *LC*-traps. There is at least one *LC*-trap involved in each of these modified filters for bypassing or blocking selected harmonics. In general, the switching frequency and its multiples are the central amounts of switching harmonics. When these frequencies are matched to the *LC*-traps resonant frequencies, PWM harmonics may be significantly reduced. The inductance of trap inductors may also be highly tiny since the resonance frequency of *LC*-traps typically matches or exceeds the switching frequency, which further reduces the overall inductance. In addition, the switching harmonic currents of multilevel converters may also be considerably reduced by using filters with small inductances^31^.

The *LTL* filters, which are high-order trapped filters, have received significant attention. Parallel *LC* traps provide for improved harmonic attenuation. However, this may lead to increased trap inductances in these filters. According to actual applications, two *LC* traps are preferable since the prominent harmonics are concentrated around the switching frequency and its first multiple. However, it is hard to minimize the output filter because these filters still require two additional inductors compared to *LCL* filters, which occupy most of the volume in filter components. It is generally important to consider the size of a filter when designing it, as the size of the filter can impact its performance and overall effectiveness. In many cases, filter design aims to achieve the desired performance level while minimizing the filter's size as much as possible. There are several reasons why the size of a filter may be an important consideration, like cost, space constraints, weight, and power density.

Most past research has focused on reducing power filters' overall inductance to lower their expense and size. The total size of the power filters is primarily due to the magnetic cores. However, high-order trapped filters include extra magnetic cores that cause a significant increment in the system size, despite a considerable reduction in overall inductance. The most frequent solution for that problem is the magnetic integration approach, which integrates many discrete inductors onto a single magnetic core^11,32–38^. To increase the converter efficiency, an EIE-type core has been proposed in^37,38^ to magnetically integrate the *LCL* filter to minimize the core size. Three-phase *LCL* filters are presented in^39^ to be magnetically integrated using a delta-yoke composite core. This core is smaller than an EIE-type core by nearly 10%. Although decoupled core configurations like EIE and delta-yoke types are possible, magnetic integration always leads to magnetic coupling. The harmonic absorption of *LCL* filters is affected by the magnetic coupling, which introduces a coupling inductance into the filter capacitor branch. In the EIE-type magnetic core,^40,41^ used a good-permeable I-type core to reduce magnetic coupling impacts.

On the other hand,^42,43^ formed an *LLCL* filter based on the magnetic coupling inductance produced through the magnetic coupling. Nevertheless, a particular air gap must be placed into the shared flux channel of an EE-type core to get the appropriate trap inductor, which limits the design flexibility and causes parameter modification to be complicated after fabrication. Since^42,43^ only achieved one *LC*-trap, the filter has a low harmonic suppression capability. In^44^, an integrated doubled-trap *LTL* filter has been presented. However, it required an extra inductor between the two *LC* traps, air gaps at the side limbs, and a complex design.

A single-phase double-trap *LCL* (*DTLCL*)-type PWM inverter for HSTs is designed using the expertise gained from the network-tied rectifiers and filters of renewable energy technologies. The *DTLCL* filter consists of two traps, one with zero impedance at the resonant frequency and one with a large impedance at the resonant frequency, in addition to the inverter-side inductance. The traps in the proposed filter attenuate harmonics at the switching and double switching frequencies, which dominate current harmonics. So, since the filter tackles lower harmonics in the higher multiples of the switching frequency, the needed filter inductors may be lowered. To reduce the high-frequency harmonics, which could cause resonance in the TPSS, this filter is designed to take the place of the traditional *L*-type and *LCL*-type rectifiers.

The area required by the *LCL* filter also has to be reduced because of the space restriction of air-core inductors in HSTs, which is solved by the presented filter. This filter would meet the power quality criteria and be well-suited for the constrained spaces in locomotives, according to simulation and hardware-in-the-loop (HIL) testing results. The magnetic core is coiled with the inductors of the filter. An easy inductor design, high inductor linearity, and simplified implementation are advantages of the presented technique.

The proposed filter will be evaluated against traditional passive filters, specifically its corresponding discrete *DTLCL* (or *SPRLCL*), *LCL*, and *L* filters, to demonstrate its viability. Only three different passive filters, *L*, *LCL*, and integrated filtering inductors *LCL* filters^8,11,45^, were utilized to reduce harmonics in traction systems, based on the authors' experience. So, the comparison is limited to the designed filter and the *LCL* and *L* filters. Furthermore, comparing the integrated *DTLCL* filter to the discrete *DTLCL* one will be done to verify the effectiveness of magnetic integrated elements in the filter design.

Additionally, the proposed methodology is compared to the SHE technique^46,47^, which utilizes a control system to suppress specific harmonics in the voltage waveform without the need for a resonant trap. However, SHE is limited to offline computations and needs extensive lookup tables at low fundamental frequencies, as well as amplifying higher-order harmonics to eliminate lower-order ones^31,48–50^. In contrast, the proposed technique is relatively easy, capable of achieving similar results at low harmonics, and reduces switching losses while eliminating harmonic distortions and improving output waveforms. It should be noted that while SHE targets voltage harmonics, the proposed approach primarily addresses current harmonics, which indirectly enhances voltage waveform quality due to the intrinsic relationship between current and voltage in electrical systems. Furthermore, the *LCL* filter capacitor and the inductance generated by the coupling effect between the two inductors create a trap without the need for additional elements, and the utilization of one magnetic core for both inductors results in a smaller overall size and associated cost savings.

The contributions of this paper are listed below:

1. An integrated double-trap filter is designed, simulated, and validated.
2. A detailed analysis of the many inductors for magnetic integration has been presented.
3. The performance of the harmonics removal and size reduction has been utilized to validate the proposed methodology.
4. The application, which is, in this case, railway traction converters, can be extended to other grid-connected inverters/rectifiers, including industrial power systems, renewable energy systems, and building power systems.
5. Identification of the potential to integrate the *DTLCL* filter directly into transformers, suggesting a promising direction for future research to enhance system integration and efficiency.

In this paper, following the introduction, the basic theory and operation characteristics of the proposed integrated *DTLCL* filter are introduced in “System structure and magnetic circuit analysis”. “Designing and modeling of magnetic integrated *DTLCL* filter” presents the magnetic integration approach, then the optimal design method to reduce filter inductances is proposed. Simulation and HIL experimental results to verify the feasibility and effectiveness of the proposed design are described in “Simulation and HIL experimental validation”. Lastly, “Conclusion” concludes the paper.

## System structure and magnetic circuit analysis

### System structure

**Figure 1:**
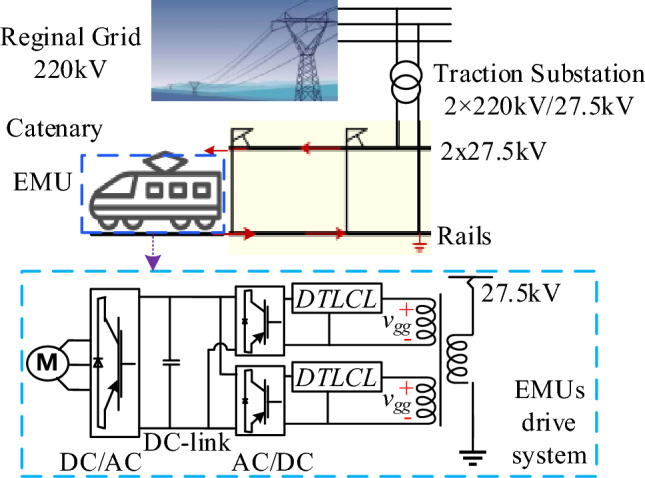
Traction grid configuration of the power supply substation with EMU.

The standard TPSS arrangement utilized in China's HSR is shown in Fig. 1^8^. To provide the all-parallel autotransformer (AT)-fed network, the three-phase 220 kV network voltage is stepped down to 27.5 kV single-phase double-feeders within the power supply station. TTs could be connected using single-phase, V/v(V/x), Ynd11, and Scott, among other methods. The V/x-structure TT is used by Chinese and German HSR due to its excellent capacity utilization, easy wiring, and compliance with AT catenary systems^1^. The all-parallel AT-fed TPSS scheme is commonly used in HSR and massive trains throughout the world due to its advantages of extended power supply, minimal voltage drop, and electromagnetic compatibility^32^. The ATs, mounted at the AT substation or section post, are separated along the track by roughly 10 to 15 km. In the complex AT traction electricity network, the feeders, contact lines, protection lines, communications cables, rail, and integrated grounding cables are all considered multiconductor transmission lines.

Figure 1 depicts an equivalent single-phase inverter driving setup for HST. The line-side converter at every HST's electric grid is made up of two PWM converters connected in parallel. Two interleaved single-phase two-level rectifiers are used in the power conversion step in Fig. 1 to reach a good current carrying capacity and power factor. As shown in^1^, the train converter is frequently two-level, several interlaced, two- or three-level. The whole paper uses two interleaved two-level PWM inverters and grid configurations of an all-parallel AT-fed EMU drive unit for analysis. There are, however, several other grid configurations and EMU kinds, as was already indicated. However, the results of the simulation and HIL experiments and the established rule of the researched filter apply to those other configurations, which opens up the possibility of multiple distinct lines of future study.

As this paper mainly focuses on the application of the magnetic integrated *DTLCL* filter, the detailed mathematical model of HSR and initial parameters are not shown here, which has previously been explored in research in^1,2,5,8,51–53^. For example, using the nodal admittance matrix, a uniform mathematical model of TPSS and China HST is presented in^51^. The HSR modeling can potentially affect the filtering effect of the integrated *DTLCL* filter. The modeling of the HSR system, including the power converters, power cables, and load, can affect the performance of the integrated *DTLCL* filter in several ways. For example, the impedance of the load and the power cables can influence the filter's resonant frequency, affecting its ability to attenuate harmonics.

Additionally, the type and level of nonlinearities presented in the HSR system can affect the harmonic content of the signals transmitted through the filter, which can also impact its performance. As described in Fig. 1, the integrated *DTLCL* filter is placed in the PWM-controlled train to suppress the harmonic generated by the traction converter since it is the origin of harmonics in the low voltage side of the TT. Therefore, the traction grid is seen from the filter as a typical linear element, with *v*_*gg*_ representing the contact-line voltage in the train location, while the equivalent load is seen as a fixed resistance^2,8,11,53^. Thus, to simplify the analysis, the values of the EMUs' converter parameters affecting the filter design and performance are listed in “Designing and modeling of magnetic integrated *DTLCL* filter”. The different grid modeling affects the designed parameters and filtering effect of the integrated *DTLCL* filter. However, the design concept and work principle are the same. This analysis is also applicable to the other passive filters.

**Figure 2:**
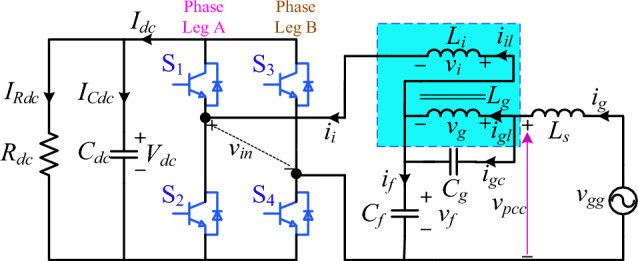
Single-phase H-bridge PWM traction inverter with an integrated *DTLCL* filter.

The harmonics assessment circuit illustration from the line-side traction converter to the traction electrical network is shown in Fig. 2. It is filtered by an integrated *DTLCL* filter. Here, a single integrated *DTLCL* converter is used to represent a typical single H-bridge configuration for analysis's sake. This approach is justified by the similarity and equivalent configuration of the two H-bridges in the system, allowing accurately predicting the harmonics reduction potential of the *DTLCL* filter in traction converters. Moreover, this research focuses on assessing the effectiveness of the magnetic integration of the *DTLCL* filter specifically in traction converter applications. Thus, by modeling only one of the similar H-bridges, it is aimed to streamline the analysis while ensuring that the findings are representative and applicable to both bridges given their equivalency. With a transformation ratio of 27.5/1.55, the TT may be regarded as a standard linear unit. The equivalent leakage inductor of the secondary side of the TT is represented by *L*_*s*_ as well. It needs to be noticed that this exact setup does not significantly limit the installation of trap filters. These filters may often be used in dc/ac or ac/dc generation systems, single- or three-phase, and standalone or grid-connected^36^. In this case, *C*_*dc*_ stands for the dc-link capacitor, *R*_*dc*_ for the equivalent load of a single integrated *DTLCL*-type rectifier on the traction inverter-motor drive system, and *I*_*Rdc*_ and *I*_*Cdc*_ for their currents. Four switches designated as S1–S4 that operate at *f*_*sw*_ transform the dc-link voltage *V*_*dc*_ into ac voltage *v*_*in*_ that contains harmonics at the dominating switching frequency 2*f*_*sw*_ and its multiples^54^. The harmonics are concentrated around 2*f*_*sw*_ when using a unipolar sinusoidal PWM (SPWM). Thus, 2*f*_*sw*_ is the actual switching frequency. It is also important to remember that harmonics at the double and fourfold switching frequencies 2*f*_*sw*_ and 4*f*_*sw*_ are much more prominent than those at high switching frequencies, which have the most output current harmonics. For this reason, the total harmonics can be effectively decreased by eliminating the harmonics at 2*f*_sw_ and 4*f*_sw_. In real situations, two *LC*-traps are favoured due to their lower expense and size, and this is the approach that is further investigated in this work. The TT might be viewed as a typical transformer. For creating the worst instability situation, the equivalent series resistors of the filter components are disregarded.

The inverter-side and grid-side inductors,* L*_*i*_ and *L*_*g*_, are connected in series. The voltages of the two inductors are *v*_*i*_ and *v*_*g*_, respectively. Additionally, *i*_*il*_ and *i*_*gl*_ are the currents flowing through the two inductors. Moreover, *i*_*gc*_ denotes the currents flowing through the capacitors *C*_*g*_. At the same time, the inverter-side current, the grid-side current, and the dc bus current are represented by *i*_*i*_,* i*_*g*_, and* I*_*dc*_, respectively. A shunt filter capacitor *C*_*f*_ has been inserted at the connection point of *L*_*i*_ and *L*_*g*_. The voltage at this point is denoted by *v*_*f*_, whereas *i*_*f*_ represents the current passing the *C*_*f*_. Moreover, *v*_*pcc*_ denotes the voltage at the point of common coupling. At this point, the inverter is tied to the grid.

The filtration effectiveness of the passive filters can be considerably impacted by the traction grid conditions. The traction grid is assumed to be weak in this article's analysis, which means that the short circuit ratio is significantly low. As a result, the grid impedance may dramatically vary^55^. For the worst case of instability, the grid might be described as a typical voltage source with series grid inductance. Since the grid resistance would reduce the resonant peaks, the focus will only be on the impact of the grid inductance, denoted by *L*_*s*_ in Fig. 2. Moreover, when many converters operate simultaneously to share power, the corresponding grid inductance detected by one converter might be proportional to the number of converters. The impact of the grid impedance gets bigger when the number of converters rises^35^. According to^43^, increased *L*_*s*_ could weaken the resonance poles. It has been discovered as a result that the filter experiences minor transient variations before going back to its setpoint. Despite this, the system's dynamic reaction required a longer time compared to the stiff grid circumstances. This paper focuses primarily on the application of the magnetic integrated *DTLCL* filter, and thus does not consider the use of *L*_*s*_ as the filter inductor of the H-bridge for estimating size reduction. This aspect has been extensively covered in previous studies, such as^11,32,56,57^, which detail the integration of filtering inductors in traction transformers for harmonic suppression. Additionally, the independence of the proposed *DTLCL* filter from *L*_*s*_ aligns with its broader applicability across various power electronics-based systems. This includes scenarios in strong grid conditions where no significant inductance is present, underscoring the versatility and robustness of the filter in diverse operational environments.

### Proposed magnetic integration approach of *DTLCL* Filter

This section will explore a detailed methodology for designing the presented magnetic integration approach of a *DTLCL* filter. The design concept of the passive filter inductors was discussed in detail throughout the relevant literature. In typical *LCL* filters, the grid-side and inverter-side inductors each have their dedicated inductor, which requires the fabrication of two inductors. Suppose two *LC*-traps are used for the *SPRLCL* filter^9^, i.e., the equivalent discrete filter to the integrated *DTLCL* one and the discrete *DTLCL* filter, as shown in “Integrated *DTLCL* filter's properties and filtering effectiveness”. In that case, it is necessary to fabricate one extra inductor and one extra capacitor in addition to the grid-side and inverter-side inductors. Thus, the discrete *SPRLCL* or *DTLCL* filter still suffers from large size and expense because of the need for one capacitor and one magnetic core for the trap inductor. This is the case even if the overall inductance is lower than typical *LCL* filters. Magnetic integration, which is an excellent technique that has previously been used in *LCL*^40,41^ and *LLCL*^42,43^ filters, is recommended for more improvement of the power density while saving expenses.

**Figure 3:**
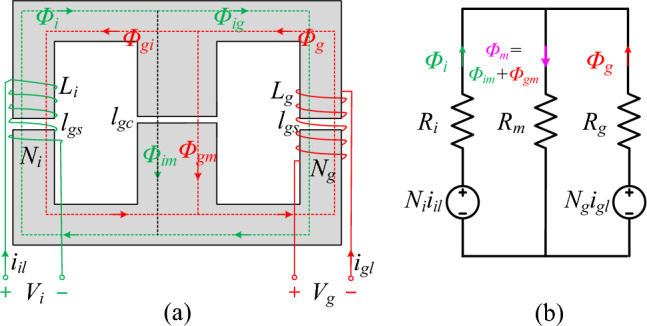
Proposed magnetic integration of *DTLCL* filter. **(a)** Core configuration of the integrated inductors and (**b)** simplified magnetic circuit.

**Figure 4:**
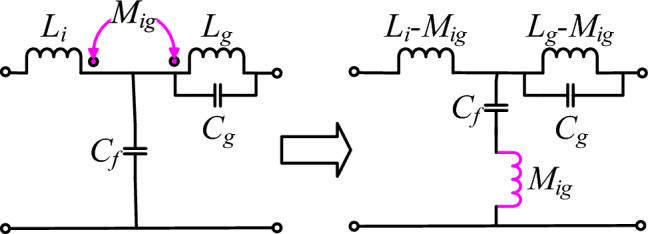
Circuit configuration of an integrated *DTLCL* filter.

Using the *LLCL* filters' magnetic integration as a basis, Fig. 3a illustrates the magnetic integration for the *DTLCL* filter, where to avoid magnetic saturation, two air gaps are introduced in the side limbs, and one air gap is also put in the central limb of a closed magnetic circuit made up of two E-type magnetic cores. Additionally, the central limb's cross-section area is double that of the side limbs to prevent magnetic saturation. In reality, E-type cores with these sizes have often been produced for industrial purposes. The main parameters for building the filter inductor are, in general, the size and material of the magnetic core, the number of turns *N*_*i*_ and *N*_*g*_, and the lengths of the central and side limbs air gaps *l*_*gc*_ and *l*_*gs*_. Air gaps can be included to prevent magnetic saturation, although doing so reduces the magnetic permeability effectiveness, necessitating a large number of turns to produce the appropriate inductances. The magnetic circuit model is shown in Fig. 3b, but the reluctances of the yokes and limbs of the magnetic core are ignored since, in the magnetic circuit, the air-gap reluctances are so much bigger than the other ones. *R*_*i*_, *R*_*m*_, and *R*_*g*_, i.e., the magnetic resistance of the three limbs, may therefore be expressed more simply as functions of the air-gap reluctances. It is necessary to assess the flux density to design the magnetic core of the integrated filter inductors. Figure 4 depicts the electric connection schematic for such a circuit. By winding *L*_*i*_ and *L*_*g*_ on the side limbs with *N*_*i*_ and *N*_*g*_, the coupling influence may be fully used to lower the filter size. In such a magnetic core, the *L*_*i*_ and *L*_*g*_ windings are negatively coupled because the flux directions at the side limbs are opposite. The central concept of the presented magnetic integration is constructing a trap-inductor via the magnetic coupling between *L*_*i*_ and *L*_*g*_ inductors called an active trap inductor.

In contrast, the other trap is formed by connecting a shunt capacitor *C*_*g*_ with *L*_*g*_ winding. In the proposed design, integrating *L*_*i*_ and *L*_*g*_ introduces a coupling inductor for one of the two *LC* traps. Based on Fig. 3b, *Φ*_*i*_ refers to the flux generated by *L*_*i*_ winding. In contrast, *Φ*_*im*_ and *Φ*_*ig*_ are the fluxes generated by *L*_*i*_ winding and flowing across the central limb and *L*_*g*_ winding, respectively, as described in Eq. (1), where the magnetic resistors of the three limbs can be referred to by *R*_*i*_, *R*_*m*_, and *R*_*g*_, respectively^35,36,43,44^.

1 $$\left\{ \begin{gathered} \Phi_{i} = \frac{{N_{i} i_{il} \left( {R_{m} + R_{g} } \right)}}{{R_{i} R_{m} + R_{i} R_{g} + R_{m} R_{g} }}, \hfill \\ \Phi_{im} = \frac{{N_{i} i_{il} R_{g} }}{{R_{i} R_{m} + R_{i} R_{g} + R_{m} R_{g} }}, \hfill \\ \Phi_{ig} = \frac{{N_{i} i_{il} R_{m} }}{{R_{i} R_{m} + R_{i} R_{g} + R_{m} R_{g} }}. \hfill \\ \end{gathered} \right.$$

*L*_*g*_ winding also generates the flux *Φ*_*g*_, where the fluxes generated by *L*_*g*_ and flowing across the central limb and *L*_*i*_ winding are *Φ*_*gm*_ and *Φ*_*gi*_, respectively, as described in Eq. (2)^35,36,43,44^.

2 $$\left\{ \begin{gathered} \Phi_{g} = \frac{{N_{g} i_{gl} \left( {R_{i} + R_{m} } \right)}}{{R_{i} R_{m} + R_{i} R_{g} + R_{m} R_{g} }}, \hfill \\ \Phi_{gm} = \frac{{N_{g} i_{gl} R_{i} }}{{R_{i} R_{m} + R_{i} R_{g} + R_{m} R_{g} }}, \hfill \\ \Phi_{gi} = \frac{{N_{g} i_{gl} R_{m} }}{{R_{i} R_{m} + R_{i} R_{g} + R_{m} R_{g} }}. \hfill \\ \end{gathered} \right.$$

Every inductor winding's total flux consists of the self-flux and the mutual flux. Then, Eq. (3) might be used to define *V*_*i*_ and *V*_*g*_^43,44^. Moreover, they can be described, according to Eqs. (1)–(3), as in Eq. (4)^36,43^, where the self-inductances *L*_*i*_ and *L*_*g*_ and the mutual inductances *M*_*ig*_ and *M*_*gi*_ could be described as in Eq. (5)^35,36,42,43^.

As a note, *M*_*ig*_ and *M*_*gi*_ are identical, and both could be written as *M*_*ig*_. It is apparent that the two inductors are coupling according to Eqs. (4) and (5), and the following could represent *V*_*i*_ and *V*_*g*_^42,43^.

3 $$\left\{ \begin{gathered} V_{i} = N_{i} \frac{d}{dt}(\Phi_{i} - \Phi_{gi} ), \hfill \\ V_{g} = N_{g} \frac{d}{dt}(\Phi_{g} - \Phi_{ig} ). \hfill \\ \end{gathered} \right.$$

4 $$\left( \begin{gathered} V_{i} \hfill \\ V_{g} \hfill \\ \end{gathered} \right) = \left( {\begin{array}{*{20}c} {L_{i} } & { - M_{ig} } \\ { - M_{gi} } & {L_{g} } \\ \end{array} } \right)\left( \begin{gathered} \frac{{di_{il} }}{dt} \hfill \\ \frac{{di_{gl} }}{dt} \hfill \\ \end{gathered} \right),$$

5 $$\left\{ \begin{gathered} L_{i} = \frac{{N_{i}^{2} \left( {R_{g} + R_{m} } \right)}}{{R_{i} R_{m} + R_{i} R_{g} + R_{m} R_{g} }}, \hfill \\ L_{g} = \frac{{N_{g}^{2} \left( {R_{i} + R_{m} } \right)}}{{R_{i} R_{m} + R_{i} R_{g} + R_{m} R_{g} }}, \hfill \\ M_{ig} = M_{gi} = \frac{{N_{i} N_{g} R_{m} }}{{R_{i} R_{m} + R_{i} R_{g} + R_{m} R_{g} }}. \hfill \\ \end{gathered} \right.$$

6 $$\left\{ \begin{gathered} V_{i} = V_{in} - V_{f} = L_{i} \frac{{di_{il} }}{dt} - M_{ig} \frac{{di_{gl} }}{dt}, \hfill \\ V_{g} = V_{f} - V_{pcc} = L_{g} \frac{{di_{gl} }}{dt} - M_{ig} \frac{{di_{il} }}{dt}. \hfill \\ \end{gathered} \right.$$

Based on Eq. (5), the self and mutual inductances are determined by the magnetic resistances that could be defined by Eq. (7). In this equation, *l*_gs_ and *l*_gc_ denote the side and central limbs' air gap lengths, and *A*_*S*_ and *A*_*C*_ represent the side and central limbs' cross-section areas, with *A*_S_ = 1/2*A*_C_. Moreover, *μ*_0_ = 4*π* × 10^−7^ N/A^2^ refers to the air permeability ^35,36,42–44^. In the end, the self and mutual inductances are determined with Eq. (8) by putting Eq. (7) in Eq. (5)^35,36,43^. As illustrated in Eq. (9), the coupling coefficient *k*_*Mig*_ is a metric that quantifies the degree of mutual inductance between two coupled circuits represented as the ratio of the mutual inductance to the square root of the product of the self-inductances. Furthermore, it can be found that the coupling coefficient could be effectively tuned by varying the air gap lengths *l*_*gc*_ and *l*_*gs*_, or their ratio *l*_*gc*_/*l*_*gs*_.

The proposed approach can accomplish the formation of one equivalent trap inductor by using the magnetic coupling between two windings, as seen in “Integrated *DTLCL* filter's properties and filtering effectiveness”. Consequently, it is possible to avoid needing two magnetic cores and decrease the expense and size of the *DTLCL* filter. Furthermore, it is observed that, in contrast to the discrete *SPRLCL* filter, there is no extra component.

7 $$\left\{ \begin{gathered} R_{i} = R_{g} = \frac{{l_{gs} }}{{A_{S} \mu_{0} }}, \hfill \\ R_{m} = \frac{{l_{gc} }}{{A_{C} \mu_{0} }}. \hfill \\ \end{gathered} \right.$$

8 $$\left\{ \begin{gathered} L_{i} = \frac{{N_{i}^{2} \mu_{0} \left( {l_{gc} A_{S} + l_{gs} A_{C} } \right)}}{{2l_{gs} \left( {l_{gc} + l_{gs} } \right)}}, \hfill \\ L_{g} = \frac{{N_{g}^{2} \mu_{0} \left( {l_{gc} A_{S} + l_{gs} A_{C} } \right)}}{{2l_{gs} \left( {l_{gc} + l_{gs} } \right)}}, \hfill \\ M_{ig} = \frac{{N_{i} N_{g} \mu_{0} l_{gc} A_{S} }}{{2l_{gs} \left( {l_{gc} + l_{gs} } \right)}}. \hfill \\ \end{gathered} \right.$$

9 $$k_{Mig} = \frac{{M_{ig} }}{{\sqrt {L_{i} L_{g} } }} = \frac{1}{{1 + 2l_{gs} /l_{gc} }}.$$

### Integrated *DTLCL* filter's properties and filtering effectiveness

Figure 4 depicts the circuit structure of the integrated *DTLCL* filter, consisting of an inverter-side inductance *L*_*i*_, in addition to two *LC* traps. These two *LC*‐traps (one is *M*_*ig*_–*C*_*f*_ and the other is *L*_*g*_–*C*_*g*_) are resonated to the dominant switching frequency 2*f*_*sw*_ and its first multiple 4*f*_*sw*_ because the harmonics are concentrated around 2*f*_*sw*_ when using a unipolar sinusoidal PWM (SPWM), where, 2*f*_*sw*_ is the actual switching frequency. The mutual inductor *M*_*ig*_ is used in the integrated *DTLCL* filter for building a resonant trap with *C*_*f*_. In contrast, *L*_*g*_ is used for creating another resonant trap with *C*_*g*_ for attenuating the switching harmonics. The resonance tanks eliminate the specific harmonics of the output current. At the same time, the other harmonics are attenuated by the whole filter. This paper proposes a method to design the integrated *DTLCL* filter by tuning the series resonant frequency to 2*f*_*sw*_ and the parallel resonant frequency to 4*f*_*sw*_.

**Figure 5:**
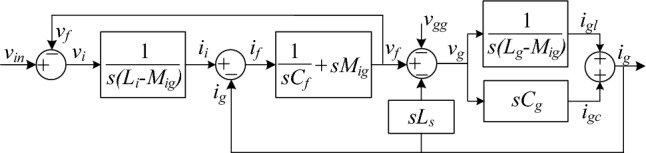
Block diagram of an integrated *DTLCL* filter.

Moreover, the block diagram of the proposed filter is presented in Fig. 5. The transfer function *G*_*DTLCL*_(*s*) of the integrated *DTLCL* filter, from *v*_in_ to *i*_*g*_, is calculated in Eq. (10), where the coefficients can be found in the Suppl. Appendix.

10 $$G_{DTLCL} (s) = \frac{{i_{g} (s)}}{{v_{in} (s)}} = \frac{{a_{4} s^{4} + a_{2} s^{2} + 1}}{{b_{5} s^{5} + b_{3} s^{3} + b_{1} s}}$$

The magnetic coupling is a key factor that has a great influence on the performance of the filter. According to Eq. (10), the mutual inductance is critical in calculating the transfer function for the overall system and, consequently, the frequency of the resonances and traps. By adjusting the mutual inductances, in other words, by tuning the coupling coefficients, these frequencies are effectively adjusted. Moreover, Eq. (9) demonstrates that the coupling coefficient may be effectively adjusted by changing the ratio *l*_*gs*_*/l*_*gc*_. Therefore, this ratio affects the transfer function of the whole plant, where this ratio is changed from around zero, i.e., *l*_gs_ ≈ 0, to about infinity, i.e., *l*_gc_ = 0. According to Eq. (8), it should be noted that *l*_gs_ cannot be zero, but it can just be very little relative to *l*_gc_, while *l*_gc_ can be zero. It can be stated that *k*_*Mig*_ values are inversely proportional to the *l*_*gs*_*/l*_*gc*_ values. These values are changed from around one to about zero.

From Eq. (10), it can be found that *G*_*DTLCL*_(*s*) has two zeros at *ω*_*t*1_ = (1/(*C*_*f*_*M*_*ig*_))^1/2^ and *ω*_*t*2_ = (1/(*C*_*g*_*L*_*g*_))^1/2^, respectively, where *ω*_*t*1_ and *ω*_*t*2_ represent the trap angular frequencies. The corresponding switching harmonics may be efficiently suppressed by setting those two frequencies to the dominant switching frequency, as indicated in Eqs. (11) and (12).‎ Moreover, the effectiveness of the proposed integrated *DTLCL* filter in reducing total harmonic distortion (THD) will be substantiated through simulation and HIL experimentation, as outlined in “Simulation and HIL experimental validation”.

11 $$f_{t1} = 2f_{sw} = \frac{1}{2\pi }\sqrt {\frac{1}{{C_{f} M_{ig} }}}$$

12 $$f_{t2} = 4f_{sw} = \frac{1}{2\pi }\sqrt {\frac{1}{{C_{g} (L_{g} - M_{ig} )}}}$$

**Figure 6:**
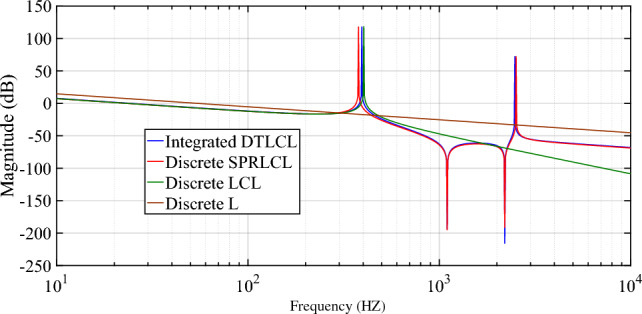
Bode diagrams for traction converters with integrated *DTLCL* and discrete *SPRLCL*, *LCL*, and *L* filters.

The Bode diagrams *i*_*g*_(*s*)/*v*_*in*_(*s*) of the integrated *DTLCL* and discrete *SPRLCL*, *LCL*, and *L* filters are shown in Fig. 6 using the parameters depicted in Table 1. Section 3.1 goes into more depth about the steps taken to design these parameters. As Fig. 6 shows, the integrated *DTLCL* filter keeps the discrete *SPRLCL* filter's features and makes a strong harmonics suppression at 2*f*_*sw*_ and 4*f*_*sw*_, where those two frequencies have two magnitude traps.

**Table 1:** Filters parameters values.

Filter type	Integrated *DTLCL*	Discrete *SPRLCL*	*LCL*	*L*
${L}_{i}({\text{mH}})$	1.63	1.63	1.63	2.93
${L}_{g}({\text{mH}})$	1.3	1.3	1.3	–
${C}_{f}(\mathrm{\mu F})$	125	125	125	–
${C}_{g} (\mathrm{\mu F})$	4.619	4.026	–	–
${L}_{f}({\text{mH}})$	–	0.167	–	–
${M}_{ig}({\text{mH}})$	0.167	–	–	–
${k}_{Mig}$	0.115	–	–	–

Furthermore, Fig. 6 shows that the magnitude-frequency characteristics of *G*_*DTLCL*_(*s*) have two conjugate resonant peaks because the degree of their denominator is 5. These resonant peaks are obtained by putting the denominator of *G*_*DTLCL*_(*s*) in Eq. (10) equals zero, substituting *s* with *jω*. The first resonFant frequency of the magnetic integrated *DTLCL* filter can be approximated as in Eq. (13)^42,43^, where *ω*_*res*1_ represents the resonant angular frequency and *f*_*res*1_ represents the resonant frequency.

13 $$f_{r1} \approx \frac{1}{2\pi }\sqrt {\frac{{L_{i} + (L_{g} + L_{s} ) - 2M_{ig} }}{{C_{f} (L_{g} + L_{s} )L_{i} - C_{f} M_{ig}^{2} }}} .$$

As Fig. 6 shows, although the discrete *LCL* filter has the largest roll-off rate of −60 dB/dec at the high-frequency domains, it has no trap magnitude, which weakens its suppression in the switching frequencies. It is essential to design the second resonant frequency far off the switching frequency multiples to prevent the harmonics amplification^26,35^ and located around 2.5 kHz, as seen in Fig. 6 . After the second resonant frequency, the proposed integrated *DTLCL* filter has a harmonics suppression of −20 dB/dec.

The resonant peaks may cause system stability problems. Several damping approaches, including passive damping^21,22,58^, and active damping^59–63^, have been proposed to ensure system stability. Designing the resonant frequency beyond the Nyquist frequency is adopted because of the additional losses of passive dampening techniques and the great sensor expenses of active dampening techniques^43,64^. In addition, the parasitic filter resistances may offer excellent dampening for enhancing system stability and performance. Other works have previously done extensive stability analysis^43,64^. Therefore, it is not included here since this chapter emphasizes the magnetic integration of the *DTLCL* filter.

## Designing and modeling of magnetic integrated *DTLCL* filter

This part of the paper will explore a detailed methodology to design the *DTLCL* filter's parameters. The ac grid is assumed to be weak in the design process in this paper, with a grid inductance of *L*_*s*_ = 4 mH. This inductance value, along with other parameter values for the EMUs inverter listed in Table 2, is derived, with slight modifications, from empirical data used in references^45,53,65^. These references report the usage of similar or even higher values of *L*_*s*​_ in comparable applications, specifically under conditions where a high series grid inductance is indispensable due to grid instability. The choice of *L*_*s*_​, although appearing elevated for general applications, is meticulously selected to align with the realistic operational scenarios our study addresses and is substantiated by literature that investigates similar conditions of grid dynamics. The magnetic integrated *DTLCL* filter parameters might be designed by implementing the system parameters listed in Table 2 in light of the regulations of the harmonic suppression meets IEEE 519–2014^8,66,67^, the consumed reactive power under 5%, and the inverter-side current ripple Δ*I*_*Li*_ less than 40%. Based on *R*_*dc*_, the inverter functions as a rectifier here. Moreover, designing *L*_*i*_, which is based on the *LCL* filter's design method in^8^, is the first step in the design process of the magnetic integrated *DTLCL* filter. As a result, *L*_*i*_ is designed as in (14) for a single-phase H-bridge unipolar SPWM inverter with Δ*I*_*Li*_ ≤ 40%, considering protecting the IGBTs and preventing saturation of the inverter-side inductor.

14 $$L_{i} = \frac{{0.5 \times 0.5 \times V_{dc} }}{{2f_{sw} \Delta I_{Li} }} \approx 1.63mH.$$

**Table 2:** Parameter values for the EMUs inverter.

Description	Symbol	Value
Contact-line voltage (RMS)	*V*_*gg*_	1550 V
DC-link capacitor	*C*_*dc*_	4 mF
Load resistor	*R*_*dc*_	10 Ω
DC-link voltage	*V*_*dc*_	3000 V
Switching frequency	*f*_*sw*_	550 Hz
Fundamental frequency	*f*_*o*_	50 Hz
Network inductor	*L*_*s*_	4 mH

Because such a system can reach the lowest possible resonance frequency to accomplish the maximum amount of inductor utilization, the *L*_*g*_ design is equivalent to the *L*_*i*_ design^37,38,64,68^. However, because the maximum current and ripple current have decreased by around 40%, *L*_*g*_ has been decreased to 1.3 mH.

To maintain the ac voltage drop in the inductors to less than 10% of the root-mean-square (RMS) value of *v*_*gg*_, the total inductance *L*_*total*_ = *L*_*i*_ + *L*_*g*_ must be monitored^9^.

When selecting the capacitor *C*_*f*_, the reactive power drawn at the fundamental frequency and harmonics elimination at high frequencies should be justified^8,69^. *C*_*f*_ may reach a small value like 125 *µ*F while taking into account the permitted reactive power.

In addition, *M*_*ig*_ is designed by satisfying Eqs. (11) and (13), i.e., *f*_*r*1_ of the magnetic integrated *DTLCL* filter is between 1/2*f*_*sw*_ and 5/6*f*_*sw*_, while *f*_*t*1_ equals 2*f*_*sw*_, i.e., *f*_*t*1_ = 1.1 kHz. To improve stability and attenuate the reduction of inductance with increasing current, *f*_*r*1_ = 2/3*f*_*sw*_ = 367 Hz was used as a midpoint value^43,64^. Consequently, *M*_*ig*_ has been calculated as 0.167 mH.

Moreover, based on Eq. (9), the relevant coupling coefficient *k*_*Mig*_ is designed as 0.115 to adjust the second resonant frequency above 4*f*_*sw*_. As a result, *l*_*gs*_/*l*_*gc*_ can be calculated to be 3.85, which proves that *l*_*gs*_ determines *L*_*i*_ and *L*_*g*_.

**Figure 7:**
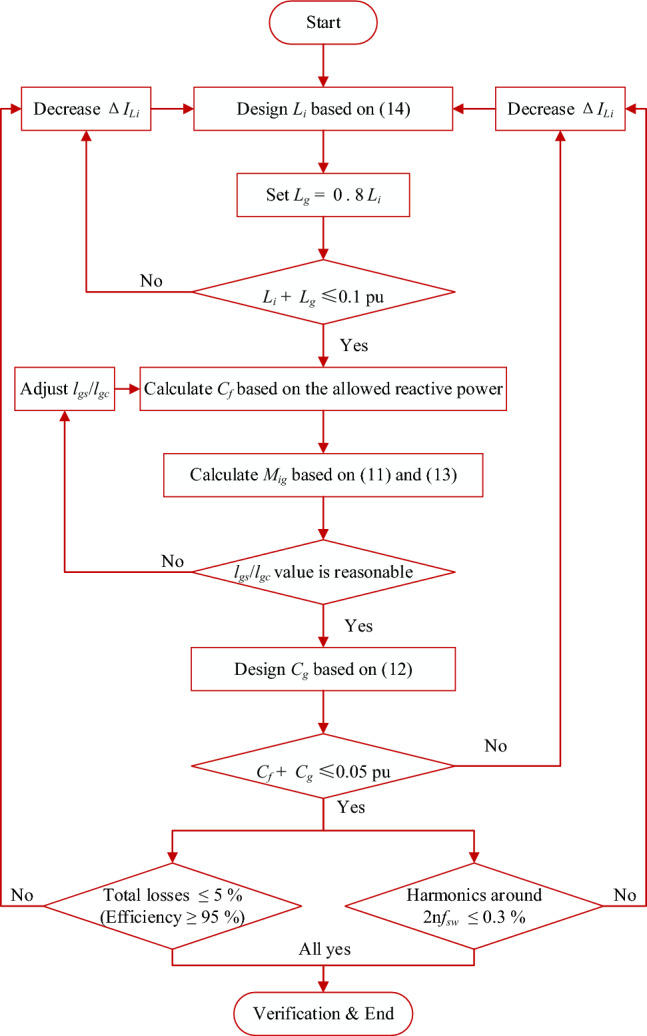
Flowchart for the proposed integrated *DTLCL* filter design method.

The additional capacitor *C*_*g*_ is determined by Eq. (12), which can be derived as 4.619 *µ*F. Nevertheless, to meet the power factor criterion, the total capacitance *C*_*total*_ = *C*_*f*_ + *C*_*g*_ must be constrained by the amount of reactive power consumed under the rated circumstances. furthermore, if *C*_*total*_ exceeded 0.05 p.u, the reactive current would be high^8,64^. The increase in filter inductance could help to solve this problem. Furthermore, Fig. 7 displays the elaborate design flowchart for the presented integrated *DTLCL* filter. This figure illustrates the flowchart of the design method as a general case, in which if one of the conditions is not fulfilled, the design process goes back to the first step. As for the inductance presented in Eq. (14) and other parameter values, they are presented as a design example to show the proposed filter design validity. In addition, according to the verification results, all the conditions are satisfied. Therefore, there is no need to go back to the first step of the design process. Furthermore, the designed parameters are slightly adjusted, and the presented ones are the final version.

In addition, the integrated inductors' power loss is minimized by using Litz wires with small ESRs and 0.5π mm^2^ cross-section area (*S*_ω_). The subsequent steps outline the design criteria for the suggested magnetic integration approach using the previously designed inductances. Choosing an EE-type magnetic core, along with its size and material, comes first. The saturation flux density *B*_sat_, relative permeability *μ*_*r*_, resistivity, and cost are typically considered when choosing a magnetic material. Higher *B*_sat_ or *μ*_*r*_ can reduce the number of turns of the inductor, which reduces the size and weight. In contrast, the eddy current losses can be lowered by higher resistivity. The magnetic material is chosen to compromise price, efficiency, size, and weight to get a cost-effective inductor. The well-known area-product approach^70^ may be used to choose the magnetic core's dimensions. The area-product (*A*_*P*_) of the cross-section and window areas (*A*_*S*_ and *A*_*W*_) of *L*_i_ is used to choose the magnetic core for the integrated windings. The EE magnetic cores are chosen depending on this technique^70^ with *L*_i_ = 1.63 mH, inductor's maximum current *I*_*il*-max_ = 800 A, and maximum flux density of magnetic core* B*_max_ = 0.35 T, where *B*_max_ is set as *λB*_sat_ with 0 < *λ* < 1, and *B*_sat_ is 0.49 T at 25 °C. *λ* is designed at 0.714 to guarantee a reasonable margin (30%). When the window area's utilization coefficient *K*_u_ = 0.5, *A*_*P*_ = 11.71 × 10^–6^ m^4^, which may be calculated by Eq. (15).

15 $$A_{P} = \frac{{L_{i} I_{il - \max } S_{\omega } }}{{k_{u} B_{\max } }}.$$

Based on the NCD products catalog^71^, a pair of E 320/160/40 cores could be chosen, with *A*_*S*_ = 1.66 × 10^–3^ m^2^ and *A*_*W*_ = 18.84 × 10^–3^ m^2^, resulting in an area-product of 31.27 × 10^–6^ m^4^ ≈ 2*A*_*P*_, which leaves a large margin of size.

The *SPRLCL* filter would be designed by following the same procedure, implying that *L*_*i*_, *L*_*g*_, and *C*_*f*_ equal those of the integrated *DTLCL* one for providing a reasonable assessment. The additional inductor *L*_*f*_ is determined by Eq. (11), replacing *M*_*ig*_ with *L*_*f*_, which is designed to be 0.167 mH. The additional capacitor *C*_*g*_ is determined by Eq. (12), with *M*_*ig*_ = 0, which can be derived as 4.026 *µ*F.

Using a similar technique, the *LCL* filter is designed, where *L*_*i*_, *L*_*g*_, and *C*_*f*_ must be comparable to those of the proposed filter to offer a reliable evaluation.

Similarly, the *L* filter is set to match the *L*_*total*_ of the proposed filter because of the same purposes or equal the *LCL* filter by setting *C*_*f*_ = 0 *μ*F.

After completing the design processes, all of the integrated *DTLCL*, discrete *SPRLCL*, *LCL*, and *L* filter parameters are provided in Table 1. In addition, the same controller implemented in^36,43,45,48,53,65,72^ may be used for the traction inverter with the integrated *DTLCL* filter, which is not discussed here. “Simulation and HIL experimental validation” will present the verification results for the proposed filter's design feasibility.

As noted, the proposed integrated double-trap filter can produce the equivalent additional inductor, thus saving one inductor with its components. In addition, the proposed magnetic integration can also save one magnetic core. This is because *L*_*i*_ and *L*_*g*_ could be wound on the side limbs of one magnetic core instead of three magnetic cores like the discrete *SPRLCL* filter and other types like *LTCL* and *LCL-LC* filters.

The comparison between the sizes of integrated inductors and discrete ones is essential. The discrete inductors of the *SPRLCL* filter could be, respectively, coiled on the middle limbs of three cores. The filter inductances of the integrated *DTLCL* filter and the *SPRLCL* one are configured to be similar to ensure adequate comparison. The discrete *SPRLCL* filter has the most considerable size with three cores. In contrast, the discrete *LCL* filter has two cores, while the integrated *DTLCL* and discrete* L* filters have one core each.

## Simulation and HIL experimental validation

To evaluate the performance of the proposed filter and design method, simulations using MATLAB/Simulink and HIL experiments were carried out on a traction inverter. The proposed filter could be validated by its comparison with the discrete *SPRLCL*, *LCL*, and *L* filters. The system's basic parameters used in the simulation and HIL experiments are described in Tables 1 and 2. With these parameters, verification models were constructed based on the system configurations shown in Fig. 2.

Analyses of the strengths and weaknesses, endurance, complexity, and size of several passive filters are conducted. The ability to attenuate harmonics and the transient performance of four filters are therefore examined using simulations and HIL testing. The several performance indexes that have been computed are listed in Table 3.

**Table 3:** Filters performance indices.

Index	Integrated *DTLCL*	Discrete *SPRLCL*	*LCL*	*L*
Size (number of cores)	1	3	2	1
DC Voltage fluctuation (V)	± 130	± 135	± 150	± 130
Power losses (kW)	22.87	23.65	24.31	N/A
Harmonics at 2*f*_*sw*_ (% *I*_*ref*_)	0.01	0.02	0.10	0.19
Harmonics at 4*f*_*sw*_ (% *I*_*ref*_)	0.00	0.02	0.03	0.12
Harmonics at 6*f*_*sw*_ (% *I*_*ref*_)	0.04	0.04	0.02	0.09
THD of *i*_*g*_ (%)	2.36	2.12	4.34	1.94

### Simulation results

**Figure 8:**
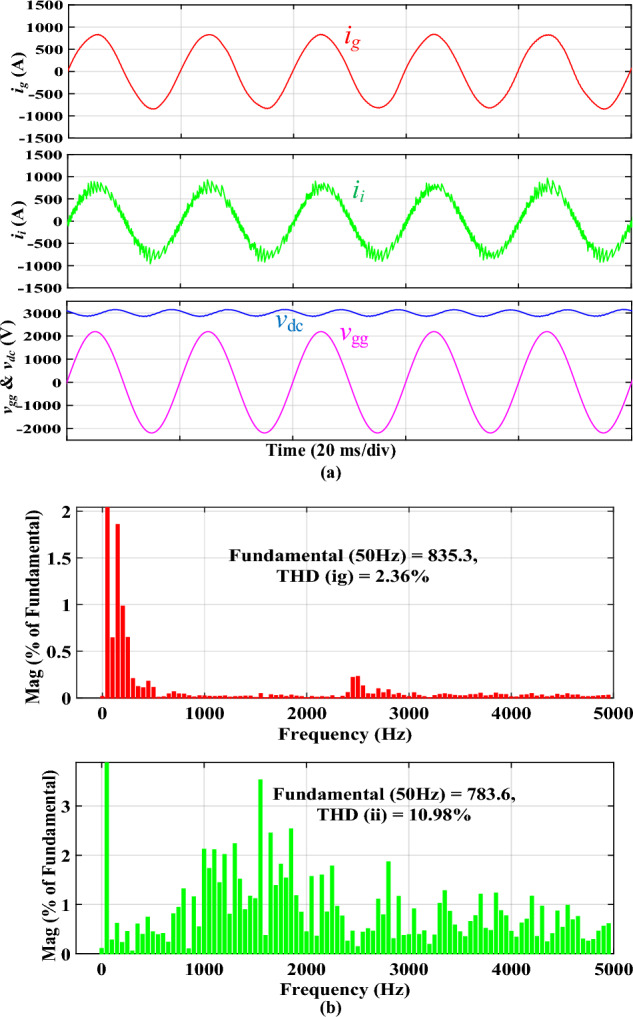
Simulated results using the integrated *DTLCL* filter. **(a)** *i*_*i*_, *i*_*g*_, *v*_*gg*_, and *V*_*dc*_ waveforms, **(b)** *i*_*g*_ and *i*_*i*_ spectra.

Figure 8a shows the simulated steady-state waveforms of *i*_*g*_, *i*_*i*_, *v*_*gg*_, and *V*_*dc*_ with the proposed integrated *DTLCL* filter. As can be seen, *V*_*dc*_ here approximates 3000 V, and the error is only 150 V (5%) owing to the control system's voltage loop. Furthermore, *i*_*g*_ is well filtered in the steady state to be hugely sinusoidal. Figure 8b shows that its THD is just 2.36%, which is very low. A significant reason for this is the proposed *DTLCL* filter's ability to attenuate low-order harmonics and the double and fourfold switching-frequency harmonics to a combined attenuation of 0.01% and 0.00%, respectively. The harmonic currents on the grid-side inductor beyond 4*f*_*sw*_ are also eliminated well, with sixfold switching-frequency harmonics of 0.04% of the fundamental current. From Fig. 8b, it is evident that there is a harmonic spike around 2.5 kHz that corresponds to the second resonance peak, confirming the theoretical analysis and Bode diagrams depicted in Fig. 6. In this case, all the harmonic currents can be limited to less than the limit of 0.3%, which complies with IEEE 519–2014 criteria. The switching harmonics composition is shown in Table 3.

**Figure 9:**
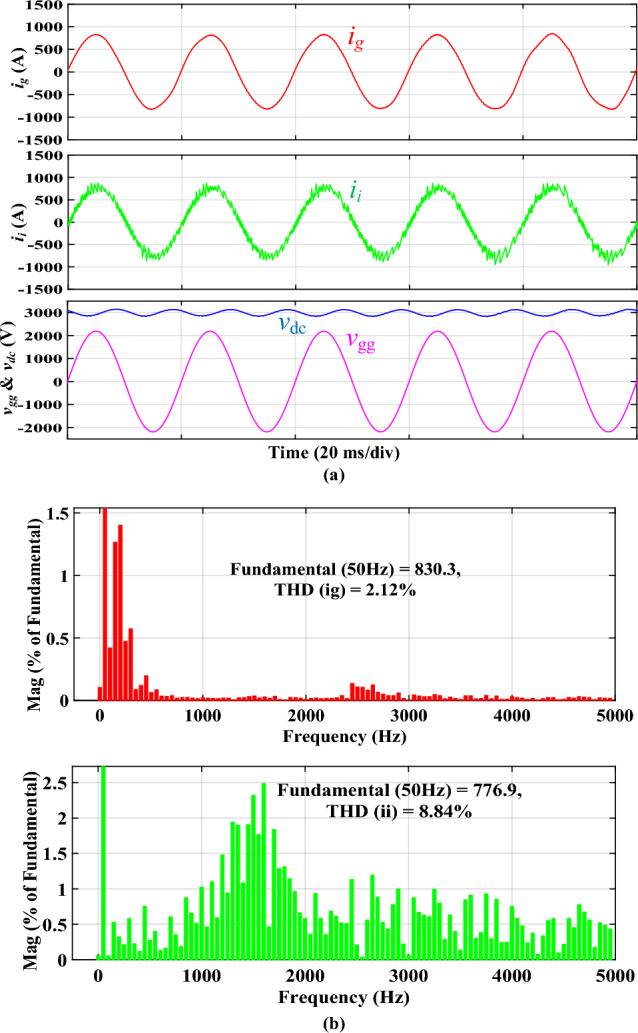
Simulated results using the discrete *SPRLCL* filter. **(a)** *i*_*i*_, *i*_*g*_, *v*_*gg*_, and *V*_*dc*_ waveforms, **(b)** *i*_*g*_ and *i*_*i*_ spectra.

Figure 9a illustrates the simulated waveforms of the traction inverter employing the discrete *SPRLCL* filter for comparative purposes. This part compares the performance of the proposed integrated filter and its discrete counterpart. Therefore, the same test has also ‎been conducted for the discrete *SPRLCL* filter, as ‎shown in Figs. 8 and 9. The waveform of *i*_*g*_ retains sinusoidal when the integrated *DTLCL* filter is substituted with the discrete *SPRLCL* filter, demonstrating that the harmonics compensating at low frequencies would not be impacted. Furthermore, as illustrated in Fig. 9b, the 2*f*_*sw*_ and 4*f*_*sw*_ harmonics of *i*_*g*_ are much reduced. The harmonics of *i*_*g*_ over the fourfold switching frequency are also efficiently suppressed. The sixfold switching-frequency harmonics are 0.04% of the fundamental component, much lower than the limits. Here, it is also apparent that there is a harmonic spike around 2.5 kHz, which confirms the theoretical analysis and Bode diagrams shown in Fig. 6. Table 3 shows that the specified *SPRLCL* filter can meet IEEE criteria with a grid-side current THD of 2.12% but with bigger filter components. As demonstrated, the suggested filter, in general, performs similarly to the discrete *SPRLCL* filter, implying its effectiveness with a small size.

**Figure 10:**
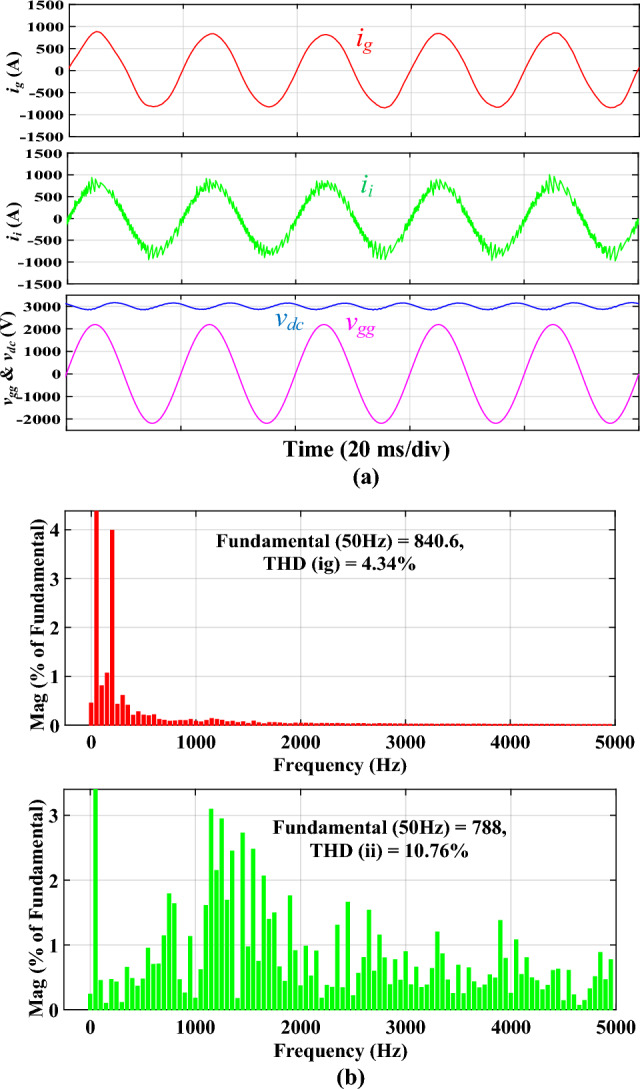
Simulated results using the discrete *LCL* filter. **(a)** *i*_*i*_, *i*_*g*_, *v*_*gg*_, and *V*_*dc*_ waveforms, **(b)** *i*_*g*_ and *i*_*i*_ spectra.

Figure 10 shows the simulated results of the traction inverter filtered by the conventional *LCL* filter. The waveforms are shown in Fig. 10a, while the spectra of *i*_*g*_ and *i*_*i*_ are shown in Fig. 10b. As depicted in Fig. 10b, the third harmonic of *i*_*g*_ exceeds the threshold of 4.00% of the fundamental component. This unipolar modulated traction inverter's somewhat inadequate performance can be attributed to its relatively low switching frequency and modest filter inductors. THD of *i*_*g*_ is 4.34%, less than the permissible level. However, the discrete *LCL* filter requires two magnetic cores compared to one for the proposed filter.

**Figure 11:**
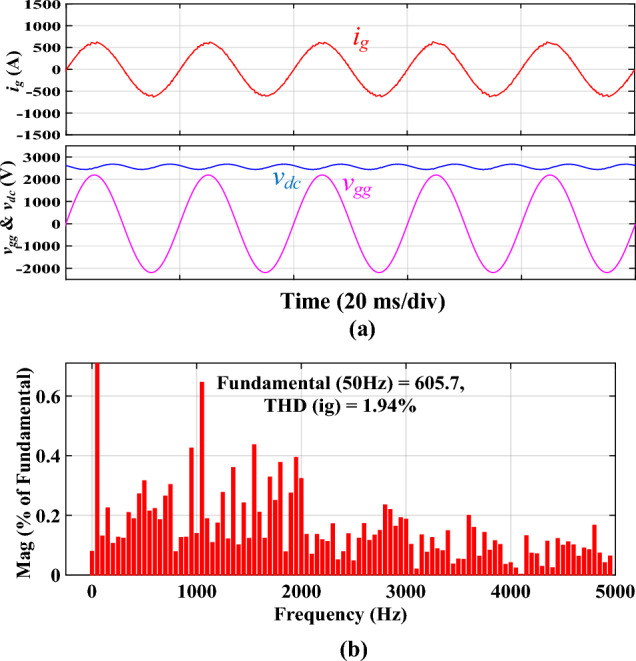
Simulated results using the discrete *L* filter. **(a)** *i*_*g*_, *v*_*gg*_, and *V*_*dc*_ waveforms, **(b)** *i*_*g*_ spectrum.

The simulated results of the traction inverter with an *L* filter are shown in Fig. 11a. Only the simulated waveform of *i*_*g*_ is shown since the waveform of *i*_*i*_ is identical. This is due to the *L* filter being a series inductance; thus, *i*_*i*_ and *i*_*g*_ are essentially the same, making it redundant to plot both current waveforms. Although *i*_*g*_ is filtered to a sinusoidal signal and in phase with *v*_*gg*_, a current spiking at the 39^th^ harmonic, near 4*f*_*sw*_, is observed, as illustrated in Fig. 11b. This occurrence is due to the *L* filter’s limited ability to attenuate high-frequency harmonics, specifically at the dominant switching frequencies such as 2*f*_*sw*_ and 4*f*_*sw*_, where the absence of a parallel *LC*-trap allows these harmonics to pass through the converter branch loop, which confirms the theoretical analysis and Bode diagrams shown in Fig. 6. As per IEEE Standard 519-2014, harmonics beyond the 35^th^ order should be reduced to less than 0.3% of the nominal fundamental current. However, the 39th harmonic reaches approximately 0.4%, thus exceeding this limit and contributing to the non-compliance with grid regulations. The grid regulations are then broken. Moreover, *V*_*dc*_ will decrease to around 2560 V, leading to inadequate inverter operation and perhaps traction stoppage. A *V*_*dc*_ of 2560 V is not permitted in reality. Hence, additional investigation into this subject is recommended.

Although this paper primarily focuses on the THDi and its implications for current harmonics, the voltage waveforms *v*_*gg*_ and *V*_*dc*_ are included to provide a comprehensive view of the system behavior under different filtering conditions. The intrinsic relationship between current and voltage in electrical systems dictates that changes in current harmonics directly affect voltage quality. Given the critical role of voltage stability in traction inverter systems, which affects operational efficacy and system reliability, maintaining voltage waveform integrity is crucial. The observed decrease in *V*_*dc*_, which can lead to traction stoppage, underscores the importance of these waveforms in the presented analysis in this paper.

From Table 3, although the THDs of the four filters, except the *L* one, are satisfied at less than 5%, the required inductance of the output filter differs. Moreover, the current switching harmonics of the first two filters at the *LC*-trap frequencies are nearly identical, proving the proposed approaches' validity. Furthermore, there are two magnetic cores saved compared to the discrete *SPRLCL* filter and one magnetic core in comparison with the discrete *LCL* one. Hence, the proposed filter reduces expenses and size.

Although the integrated *DTLCL* filter is smaller and lighter compared to the discrete *SPRLCL* and *LCL* ones, a thorough assessment must take the inductors' power loss into account. Because it is unstable, the *L* filter is not taken into consideration herein. The detailed analysis of the inductors' power losses, which has previously been explored in previous research^43^, is not included here since the primary focus of this paper is on the magnetic integration and application of the *DTLCL* filter in traction inverters. Inductors often lose power as a result of core and winding copper losses. Due to the numerous harmonics in *i*_*i*_ and *v*_*i*_, the power loss of the inductors could not be computed or measured directly. To accurately evaluate the inductors' power loss of the integrated *DTLCL*, discrete *SPRLCL*, and *LCL* ones, the system efficiency is determined under similar circumstances. When performing under a unit factor, *P*_*in*_ denotes the input active power calculated using *P*_*in*_ = *V*_*gg*_*I*_*g*rms_, while *P*_*o*_ denotes the output active power calculated using *P*_*o*_ = *V*_*dc*_*I*_*dc*_. The efficiency of the system is assessed using the ratio *P*_*o*_/*P*_*in*_. The average values of *i*_dc_ and *v*_*dc*_ that can be calculated or measured directly are *I*_dc_ and* V*_*dc*_. *I*_*g*rms_ and *V*_*gg*_ are the RMS values of *i*_*g*_ and* v*_*gg*_. The power loss is calculated by subtracting *P*_*o*_ from *P*_*in*_. The system efficiency of the integrated *DTLCL* and discrete *SPRLCL* and *LCL* ones under conditions equivalent to full load are 97.52%, 97.44%, and 97.37%, respectively. The efficiency is roughly equal to 97.3%, indicating low system power losses.

**Figure 12:**
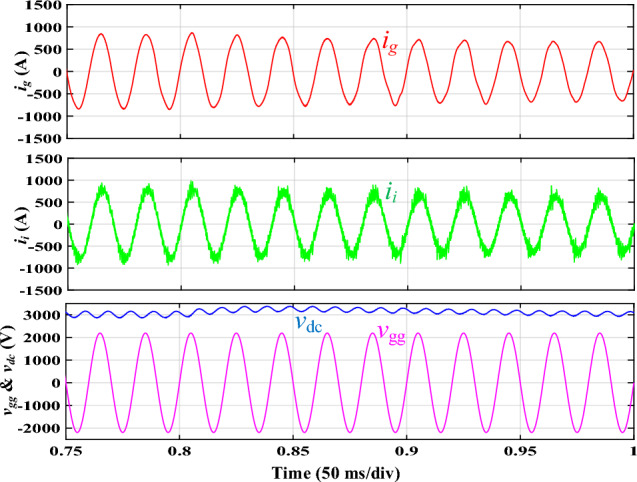
Simulated results using the integrated *DTLCL* filter with a step-up load variation of 2.5 Ω.

The load variation testing is performed on the proposed integrated *DTLCL* filter, where the related simulated waveforms are displayed in Fig. 12. At t = 0.8 s, the load was increased to 125% of its rated value (10 → 12.5 Ω). The capability of the filter to suppress instability, which is determined by going back to the setpoint, is the most crucial requirement in the transient state. As can be seen, the integrated *DTLCL* filter is strong enough to maintain stability even though the load varied by 25% in the transient duration. The dc-link voltage spike is lower than 300 V, and the total variation length was 0.12 s, followed by a smooth return to the setpoint, indicating a well-designed filter.

**Figure 13:**
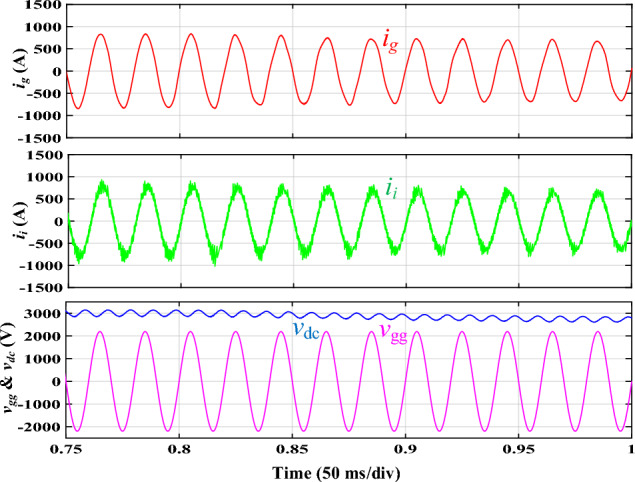
Dynamic simulated results using the integrated *DTLCL* filter.

A 300 V step-down variation in *V*_*dc*_ (3000 → 2700 V) was performed at t = 0.8 s using the proposed integrated *DTLCL* filter to check the dynamic operation, where Fig. 13 shows the simulated waveforms. It is shown that before returning to the setpoint without fluctuating, the filter experiences a few transients. The system's dynamic responsiveness required a brief time until the current started following its reference, even though the transient phase lasted only for around 80 ms. As can be seen, the stability was preserved by the integrated *DTLCL* filter. Moreover, the proposed approach still demonstrates strong switching harmonics suppression abilities during the dynamic phase.

Figures 12 and 13 show that the grid-side current is almost ringing-free. On the other hand, the system's dynamic responsiveness requests more time until the current follows the reference, leading to a longer dynamic period. According to the simulated results, because the integrated *DTLCL* filter has a sturdy construction, it is acceptable for traction networks.

### HIL experimental results

**Figure 14:**
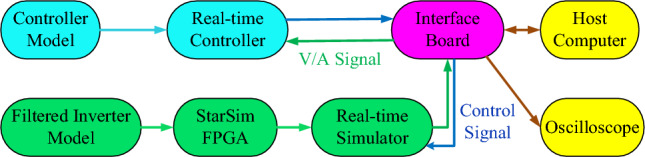
HIL experimental platform's schematic diagram.

For further verification, the experiments are also conducted on the HIL platform. The HIL experiments platform is set up to validate and test the presented filters' superiority, durability, and stability. The main advantage of the HIL experimental platform is that the real prototype may be evaluated without the need for underlying devices, as shown in Fig. 14^73^. Another advantage is that the designer need not depend on environmental or natural testing. Additionally, because the models could depict the plants, it is useful and economical. Using HIL, it is feasible to reduce the expense of physical validations in addition to the effort and time required for developing modifications in a wide range of situations^74,75^. Moreover, HIL experiments ease the recognition and redesigning barriers. In addition, it enables the real-time test to progress through the entire process more quickly than the physical test. Furthermore, HIL is preferable to physical tests in precision and expense since it can be designed and operated on a timetable.

This method has shown considerable potential for business and academics thanks to HIL's ability to provide risk-free equipment and a speedy prototyping approach in research and engineering^4,8,32,36,43,53,72–81^. In cases when an extensive system is employed, HIL experiments may offer a secure testing environment. HIL is an attractive technique that offers the capabilities to test design methodology in a scenario of extensive systems with many complicated independent models that have high switching frequencies or quick dynamic behavior^4,8,32,53,74,76,81^. Furthermore, HIL is a contemporary technique frequently utilized for power electronic system testing and validation. To address the problems of difficulties, complexities, and expense, HIL has been used to assess power inverters^73,75,77–80^. The evaluation by HIL validation of the network-tied converter with passive filters is made more desirable, according to the results in^8,32,36,43,72,77,78^.

The presented filter is currently in the research phase, which must be clarified. Rather than the physical filter design that could happen in the subsequent phases, the presented filters would be created and evaluated utilizing the HIL platform during this phase since this is more cost-efficient and it is impossible to build an actual TPSS at laboratory tests. It is essential to note that the HIL technique is a powerful validation tool for new design techniques in large systems like TPSS since, with its help, it is possible to assess the accuracy and efficiency of the investigated systems without having to spend funds for their real implementation^4,8,32,53,74,76,81^. The standard method in the HIL technique is used in this study to represent the presented filters. Utilizing this standard method for the examined circuit, it is believed that the HIL technique offers relevant results that are extremely near to those of the real tests, as in the systems presented in^8,32,36,43,72,77,78^.

**Figure 15:**
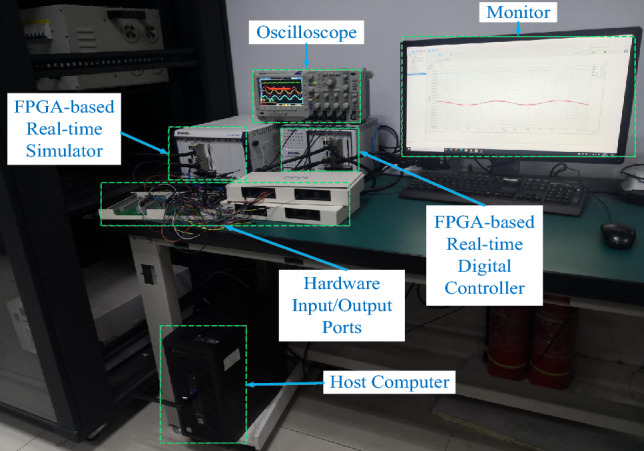
HIL experimental setup.

A fast control prototyping unit developed by StarSim modeling and integrated into the NI PXle-8821-FPGA-7868R real-time controller (RTC) and the NI PXle-8821-7846R real-time simulator (RTS) are all part of the HIL^76^, as shown in Fig. 15. The HIL also includes a power system emulating unit, hardware input/output ports, an oscilloscope, and a host computer. The translucent backboard of the NI PXle-1082 has eight slots and offers exceptional performance and output power. Moreover, HIL matches the OXI-5 PXI hardware requirements, has improved synchronization features, and delivers a high degree of reliability, leading to a low mean time for repairing^75^.

MATLAB/Simulink can be utilized for programming the control system, where the fixed-step solver can be used. Using the modeling program StarSim, the grid-connected rectifier with filter and control system models is uploaded into the HIL. Executing the grid-connected rectifier with filter model and control system model in RTS and RTC, respectively, results in constructing a closed loop. Like the simulation verification, identical parameters are used. The oscilloscope and monitor could be used for tracking the voltage and current waveforms. The oscilloscope may also provide data for the experimental current/voltage waveforms, which might then be uploaded to the MATLAB/Simulink program and examined with the Powergui FFT Analysis Tool.

**Figure 16:**
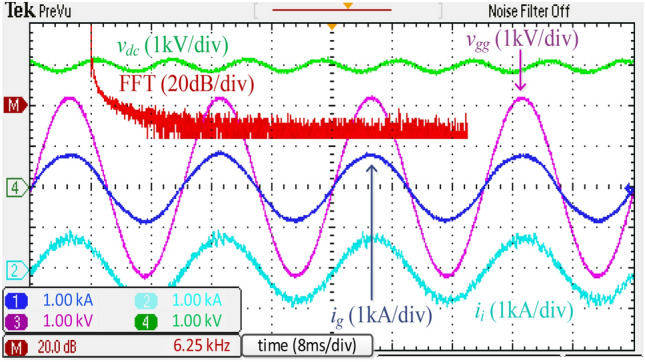
Experimental waveforms and grid-side current spectrum using the integrated *DTLCL* filter.

Figure 16 depicts the experimental waveforms with the harmonic spectrum of *i*_*g*_ of the integrated *DTLCL* filter. The controller causes *V*_*dc*_ to be close to 3000 V, and the error is only 150 V (5%). Additionally, *i*_*g*_ is appropriately filtered to be extremely sinusoidal. Because of placing the two *LC*-traps at the frequencies of 1.1 and 2.2 kHz, the current switching harmonics were significantly decreased below 0.3% of the rated current, and the system meets the IEEE 519-2014 regulations.

**Figure 17:**
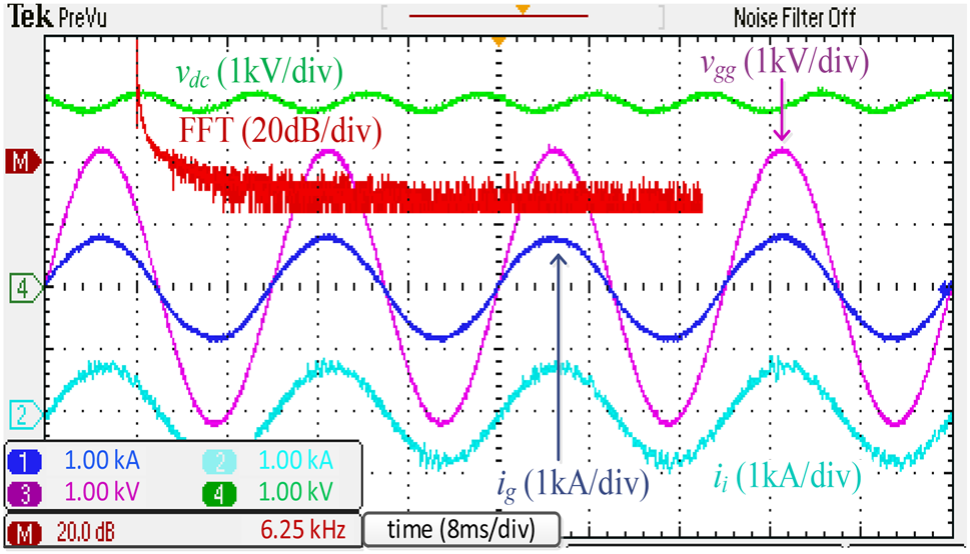
Experimental waveforms and grid-side current spectrum using the discrete *SPRLCL* filter.

For comparison, Fig. 17 shows the experimental results obtained using a discrete *SPRLCL* filter as a substitute for the proposed integrated *DTLCL* filter. The *i*_*g*_ waveform maintains its sinusoidal shape, proving that the low-order harmonics compensation is unaffected. The double and four-fold switching frequency harmonics, which are the two prominent current switching harmonics, have been effectively diminished. Compared with Fig. 16, the performance of the proposed filter is often comparable to that of the discrete *SPRLCL* one, indicating that it is effective despite being small in size.

**Figure 18:**
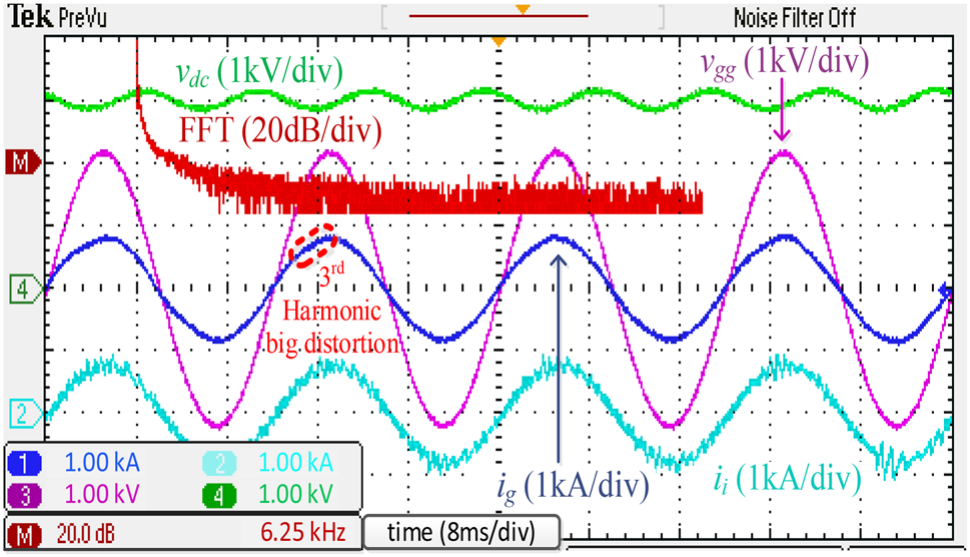
Experimental waveforms and grid-side current spectrum using the discrete *LCL* filter.

Figure 18 illustrates the experimental waveforms and *i*_*g*_ harmonic spectrum of the discrete *LCL* filter. The low-order harmonics of *i*_*g*_ were found to nearly surpass the limits. This problem is caused by the low switching frequency of the unipolar modulated traction inverter and tiny filter inductors. Like the simulated results in Fig. 10, the THD of *i*_*g*_ was seen as less than allowable levels.

**Figure 19:**
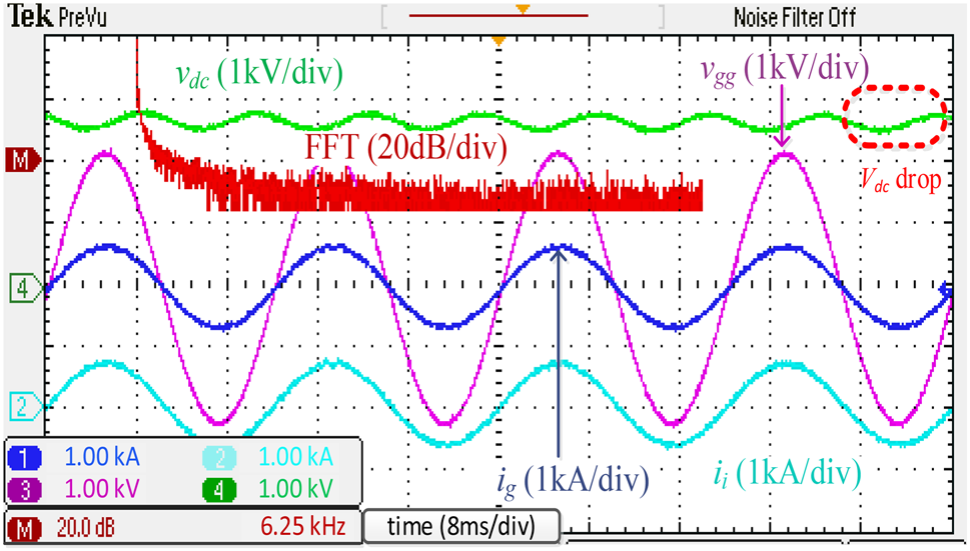
Experimental waveforms and grid-side current spectrum using the traditional *L* filter.

Figure 19 depicts the experimental results of the conventional *L* filter. Although *i*_*g*_ is filtered to a sinusoidal shape and in phase with *v*_*gg*_, a current spiking in the 39^th^ harmonic, beside 4*f*_*sw*_, was observed, which is a breach of the network regulation. Furthermore, the *V*_*dc*_ decreased to around 2560 V, like the simulated results in Fig. 11. Consequently, this situation may lead to bad inverter performance or traction blocking. A 2560 V *V*_*dc*_ must not be allowed. As a result, additional research needs to look at the problem more thoroughly.

**Figure 20:**
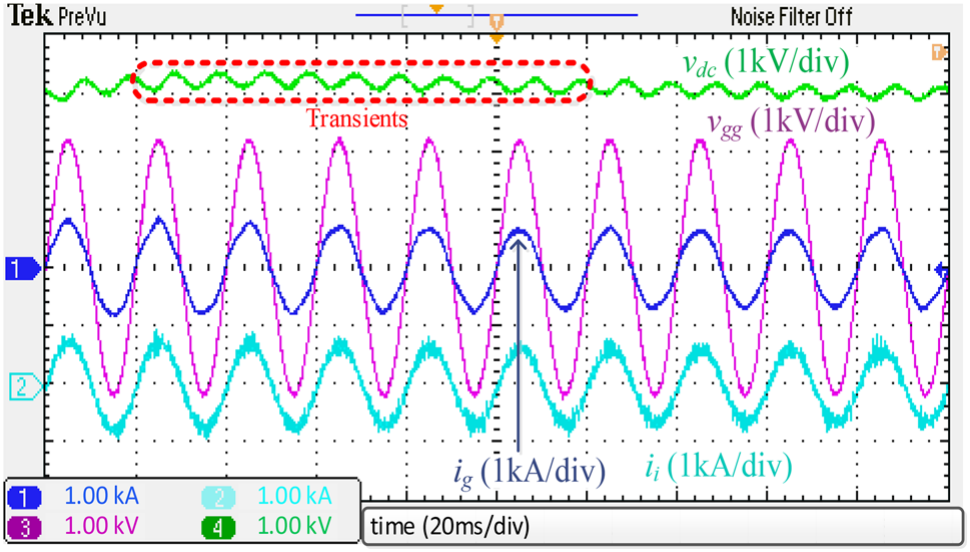
Experimental waveforms using the integrated *DTLCL* filter with 2.5Ω step-up variation to *R*_*dc*_*.*

A step variation in the dc load occurred to investigate the system's transient performance of the integrated *DTLCL* filter. Figure 20 depicts the experimental results when the load gets a step change from 10 to 12.5 Ω at 0.8 s to evaluate the proposed filter's capacity to follow commands. As shown, the system can function appropriately in the face of transient occurrences, comparable to the simulation results in Fig. 12. The essential need in the transient state is the filter's capacity to suppress instability, which is assessed by returning to the setpoint. The integrated *DTLCL* filter is powerful enough to ensure stability even when the load varies. As observed, the dc-link voltage experienced a voltage rise before progressively dropping to the setpoint.

**Figure 21:**
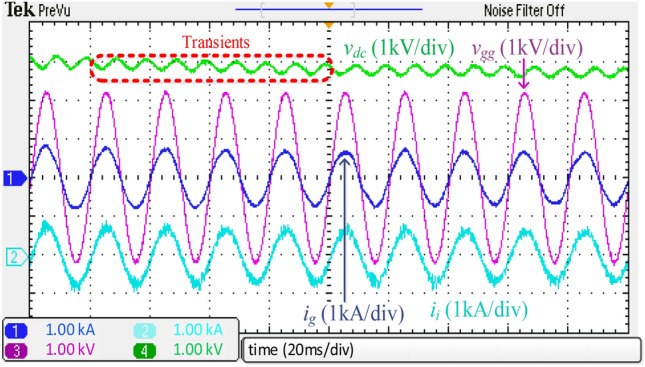
Dynamic experimental waveforms using the integrated *DTLCL* filter.

Next, the dynamic test was performed for the *DTLCL* filter, where Fig. 21 shows the experimental results of a 300 V step-down variation in the dc-link voltage. As can be observed, the waveforms are comparable to those simulated in Fig. 13, proving that the filter can provide both stability and switching harmonic attenuation. The *DTLCL* filter is seen to have certain transient moments and then return to its setpoint with no fluctuation, which verifies the system's robustness. Even though the transient phase only lasted for around 80 ms, the system's dynamic reaction needed a brief time till the current began following its reference.

All the experimental results are generally in agreement with the simulated ones and the theoretical analyses presented in the previous sections. These simulation and experiment results verify the theoretical analysis precision and confirm that the proposed integrated *DTLCL* filter keeps the advantages of the discrete *SPRLCL* and *LCL* filters and overcomes their disadvantages. The results show that the presented filter performs similarly to the discrete *SPRLCL* filter, the proposed parameter design approach is effective, and the proposed parameter robustness analysis technique is accurate. Furthermore, the integrated *DTLCL* filter has flexibility and performance under different working conditions.

## Conclusion

A magnetic integrated double-trap filter, referred to as *DTLCL,* is proposed in this paper for traction rectifiers to lessen the inductors' size and weight because the space on high-speed trains is highly constrained while suppressing the dominating current switching harmonics. Based on traditional *LCL* filters, a tiny capacitor placed in parallel with the grid-side inductor could be used for constructing an *LC*-trap. Another *LC*-trap could be created by introducing the coupling inductance into the filter capacitor branch via the magnetic coupling of the inverter-side and grid-side windings. It is possible to tune these two *LC* traps to specific harmonic frequencies using a stepwise design method. The proposed filter can achieve the same harmonic suppression performance as the discrete double-trap filter, such as the *SPRLCL* filter, and save two magnetic cores of two trap inductors.

Furthermore, the presented filter has a magnetic core structure like the integrated *LCL* one but performs better in harmonic suppression. In addition, the resonance frequency is set over the Nyquist frequency, which equals half the sampling frequency, for using this design. The presented double-trap filter has been provided with a detailed step-by-step design method to facilitate the parameter choices. The developed filter could also withstand the grid impedance changes. After Simulink simulations and HIL experimental models were completed, the verification results were provided to demonstrate that the integrated *DTLCL* filter has the following advantages:

1. It has fewer discrete passive components than the discrete *DTLCL* and *LCL* filters.
2. Compared to other conventional passive filters, it effectively suppresses harmonics.
3. Flexibility in filter design and effectiveness of magnetic integration.
4. It has durability and stability to transient and dynamic occurrences.

### Supplementary Information

Supplementary Information.

## Data Availability

The datasets generated and/or analysed during the current study are not publicly available due personal will but are available from the corresponding author on reasonable request.
