# Supplementary material for: Magnetic integrated double-trap filter utilizing the mutual inductance for reducing current harmonics in high-speed railway traction inverters
Source: Sci Rep. 2024 May 2;14:10058. doi: 10.1038/s41598-024-60877-y (PMC11065895; doi:10.1038/s41598-024-60877-y)
Supplement: Supplementary file 1 — Supplementary Information. [file 41598_2024_60877_MOESM1_ESM.docx]

1. Appendices

The following is a list of the coefficients in (10):

$$a_{4}=C_{f}C_{g}M_{ig}(L_{g}-M_{ig})$$

$$a_{2}=C_{f}M_{ig}+C_{g}(L_{g}-M_{ig})$$

$$b_{5}=C_{f}C_{g}(L_{g}-M_{ig})(L_{i}M_{ig}-M_{ig}^{2}+L_{i}L_{s})$$

$$b_{3}=C_{f}(L_{i}-M_{ig})(L_{g}-M_{ig}+L_{s})+C_{g}(L_{g}-M_{ig})(L_{i}-M_{ig}+L_{s})+C_{f}M_{ig}(L_{i}+L_{g}-2M_{ig}+L_{s})$$

$$b_{1}=L_{i}+L_{g}-2M_{ig}+L_{s}.$$
